# The Microbiology of Tonsils in Khamis Civil Hospital, Saudi Arabia

**DOI:** 10.5402/2012/813581

**Published:** 2012-10-17

**Authors:** Mohammed S. Al Ahmary, Ali S. Al Mastour, Wagih M. Ghnnam

**Affiliations:** ^1^ENT and Neck Surgery Department, Khamis Mushayte General Hospital, Saudi Arabia; ^2^General Surgery Department, Mansoura Faculty of Medicine, Egypt

## Abstract

*Objectives*. Tonsillitis is a common infection in all age groups, especially under the age of five. Organisms causing this condition vary from place to place. Our aim is to find out the main causative agents of this condition in our hospital. *Patients and Methods*. Fifty-two consenting patients who needed tonsillectomy in Khamis civil hospital, Kingdom of Saudi Arabia, between September 2006 and April 2007, were enrolled for the study. Swabs were taken from their inner surfaces and cultured for anaerobes and aerobes according to standard microbiological techniques. *Results*. Fifty-two patients, consisting of 30 males and 22 females were enrolled. Their mean age was 9.81 ± 6.47. Nearly 65% of patients had positive cultures while 35% were negative. The commonest bacteria isolated were *Staphylococcus aureus* (44.1%); and Group B *Streptococcus* (35.3 %). Two Gram-negative bacteria, *Klebsiella pneumoniae*, (8.82%), and *Pseudomonas aeruginosa* (2.94 %), were also isolated. No anaerobe was isolated. *Conclusion*. Gram-positive cocci, consisting of *Staphylococcus aureus* and Group B *Streptococcus* (*Streptococcus agalactiae*), are the major causes of tonsillitis requiring surgery in our hospital. Antibiotic treatment of this condition should be directed largely against these organisms.

## 1. Introduction

The lingual tonsils develop at 6.5 weeks between the second and third arches ventrally while palatine tonsils develop at 8 weeks from second pouch (ventral and dorsal) [1]. Tonsils are predominantly B-organs and B-lymphocytes comprise 50–60% of tonsillar lymphocytes [2]. T-cell lymphocytes comprise approximately 40% of adenoids and tonsillar lymphocytes [3]. Conversely, 70% of the lymphocytes in peripheral blood are T-cells [3]. Ample evidence shows that tonsils are involved in inducing secretory immunoglobulin production [4, 5]. Both adenoids and tonsils are favourably located to mediate immunologic protection of the upper aerodigestive tract as they are exposed to air borne antigens [2]. Tonsils are particularly designed for direct transport of foreign material from the exterior to the lymphoid cells [2]. The human tonsils are immunologically active between the ages of 4 and 10 years [2]. Involution of the tonsils begins after puberty, resulting in a decrease of the B cell population and a relative increase in the ratio of T to B cells [2]. The commonest indication for tonsillectomy is recurrent tonsillitis, which results in shedding the immunologically active cells and decreasing antigen transport function with subsequent replacement by stratified squamous epithelium [6, 7]. Recurrent tonsillitis requiring surgery is a common phenomenon in Khamis Civil Hospital. We investigated the common pathogens causing this condition in our hospital and report here our findings.

## 2. Patients and Methods

Patients presenting at our clinic with signs and symptoms of chronic tonsillitis were enrolled for the study. The study was explained to them and where children were involved, to their parents. Those who agreed were given consent form to sign.

Before the operation began, the laboratory was informed and a technician stood by to collect the tonsil as soon as it was removed. Sterile wide-mouthed container was provided and the excised tonsils were aseptically put into them and carried immediately to the laboratory for processing. As soon as the tonsil reaches the laboratory, it is cut into two with a sterile surgical blade; the inner surfaces were swabbed with sterile cotton swab, and inoculated onto two blood agar plates, one MacConkey agar and one chocolate agar plate. One blood agar plate was incubated anaerobically, the chocolate plate in 5–10% CO_2_ while the rest of the plates were incubated aerobically. The aerobic plates and the CO_2_ plate were examined after 24 hours; if no growth, they were reincubated for a further 24 hours after which if still no growth, they were discarded as negative. The anaerobic cultures were examined at 72 hours and if no growth they were reincubated for a total of 7 days. Plates showing no growth on day 7 were discarded as negative. All bacterial isolates were processed according to standard microbiological technique [8]. Sensitivity testing of isolates was done in the Micro scan machine.

## 3. Results

There were 52 patients, made up of 30 males and 22 females. Their age range is from 3–27 years, mean age 9.81 ± 6.47. There were 34 bacterial isolates from 52 patients, giving a percentage positivity of 65.38%. Thirty isolates were Gram-positive bacteria and only four were Gram-negative, made up of two genera, *Klebsiella* and *Pseudomonas*. *Staphylococcus aureus* was the predominant isolate (15/34, 44.1%), followed by group B Streptococcus (12/34, 35.3%). Others were, *Streptococcus pyogenes* (group A Streptococcus), 1/34, 2.94%; and untypable Streptococcus spp. 2/34, 5.88%. The Gram-negative bacteria consist of *Klebsiella pneumoniae* 3/34, 8.82% and *Pseudomonas aeruginosa* 1/34, 2.94% (Table 1). There were no growths in 7 patients while 11 yielded growth of normal flora only. No anaerobe was isolated. All the cases were chronic and most of them took antibiotics before presenting to us.

## 4. Discussion

Establishment of normal flora in the upper respiratory tract begins at birth [9]. The ratio of anaerobic to aerobic bacteria in saliva is approximately 10 : 1 because of variations in oxygen concentration throughout the oral cavity [10]. Invargsson et al. revealed that *Streptococcus pneumoniae* was recovered in 19% of healthy children, *Hemophilus influenzae* in 13%, group A *Streptococcus* in 5%, and *Moraxella (Branhamella) catarrhalis* in 36% [11]. The frequency of pathogens decreases with age, possibly because of increased immunity [11]. Because the oropharynx is colonized by many organisms, most infections of Wadeyer's ring are polymicrobial [12]. In our study, the predominant isolate was *Staphylococcus aureus*, accounting for 44.1%. This is in agreement with Brook et al. [13] but in contrast with other researchers who found beta-hemolytic *Streptococci* to be the predominant isolate [14, 15]. These authors also found that *Streptococcus pyogenes* was isolated more frequently in recurrent tonsillitis while in the tonsillar hypertrophy, Streptococci beta-hemolytic non A group predominated [14]. It has been suggested that fine-needle aspiration can be used in identifying tonsil core bacteriology in clinical settings [16]. Methicillin resistant *Staphylococcus aureus* (MRSA), has been isolated from the surface and core tonsils in children [17]. We did not encounter any MRSA in our study; and all the *Staphylococcus aureus* isolated were sensitive to Augmentin and Vancomycin. It would appear from our results, that Augmentin should be our drug of choice in future treatment of tonsillitis from this centre. The findings of Kuhn et al. supported the etiologic role of *Hemophilus influenzae* and *Staphylococcus aureus* in hypertrophic tonsils with or without inflammation [18, 19].

## Figures and Tables

**Table 1:** Sex distribution of bacterial isolates.

Organism	Male sex	Female sex	Total
*Staphylococcus aureus *	9	6	15
GBS	8	4	12
*Streptococcus pyogenes *	1	0	1
Untypable strep	2	0	2
*Klebsiella* sp.	0	3	3
*Pseudomonas* sp.	1	0	1
Normal flora	6	5	11
No growth	3	4	7

Total	30	22	52
